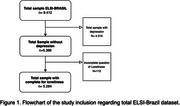# Functional independence and cognition associated with loneliness in a nationwide brazilian cohort

**DOI:** 10.1002/alz70860_105876

**Published:** 2025-12-23

**Authors:** Joana Emilia Senger, Marco De Bastiani, Lucas Uglione Da Ros, Eduardo R. Zimmer, Wyllians Vendramini Borelli

**Affiliations:** ^1^ Universidade Federal do Rio Grande do Sul, Porto Alegre, RS, Brazil; ^2^ Universidade Federal do Rio Grande do Sul, Porto Alegre, Rio Grande do Sul, Brazil; ^3^ Universidade Federal Do Rio Grade Do Sul, Porto Alegre, Rio Grande do Sul, Brazil; ^4^ Centro de Memória, Hospital Moinhos de Vento, Porto Alegre, RS, Brazil

## Abstract

**Background:**

Identifying modifiable risk factors to address the rising rates of dementia has become a priority. In this context, loneliness has emerged as a key factor associated with cognitive decline. This study aimed to examine the relationship between loneliness, social isolation, and cognitive performance, considering demographic variations in the Brazilian population.

**Method:**

Data from 5,284 participants (mean age: 62.8 ± 9.4 years; 50.1% male) aged 50 and older were obtained from ELSI‐Brazil, a population‐based study (Figure 1). Only non‐depressed participants were included. A global cognitive composite score was calculated based on cognitive assessments of immediate and delayed recall, orientation, and semantic fluency. Functional independence was measured using a modified version of the Lawton scale. To assess loneliness, participants were asked a single question: “How often do you feel lonely?” Those who responded “always” or “sometimes” were classified as experiencing loneliness. All analyses were conducted using packages in R software (V4.1.0).

**Result:**

Loneliness was a predictor of cognitive scores (beta = ‐0.07, *p* = 0.006) and functional independence (beta = 0.18, *p* = 0.0004) in all ages. When analysed adults and elderly separately, the association between loneliness and cognitive scores was significant only in older adults (beta = ‐0.09, *p* < 0.001). Loneliness was also a significant moderator of the association between age and cognition only in males (beta = ‐0.09, *p* = 0.006). The association between functional independence and age was moderated by loneliness only in males (beta = ‐0.23, *p* = 0.006). Both relationships remained significant in a sensitivity analysis after adjusting for social isolation.

**Conclusion:**

Males were particularly vulnerable to the moderating effect of loneliness in cognitive decline and aging. This suggests that the negative impact of aging on cognitive performance is more pronounced in lonely men than in non‐lonely. It is important to consider this factor with the idea that our cognitive health may require more than the factors we are already familiar with. Furthermore, understanding the population is crucial in order to target prevention policies to the appropriate groups.